# The Modality Effect on Delayed Free Recall in Non-demented Patients With Mild Parkinson’s Disease Progression

**DOI:** 10.3389/fnagi.2019.00189

**Published:** 2019-07-23

**Authors:** Constantinos Kormas, Ioannis Zalonis, Ioannis Evdokimidis, Elisabeth Kapaki, Constantin Potagas

**Affiliations:** First Department of Neurology, Aeginition Hospital, National and Kapodistrian University of Athens, Athens, Greece

**Keywords:** mild Parkinson’s disease progression, delayed recall impairment, memory modalities, rey auditory verbal learning test, rey osterrieth complex figure test, greek version of face-name associative memory examination

## Abstract

**Background**: The modality effect plays the central role in learning and memory functions. Retrieval failure constitutes a common memory impairment that occurs among patients with Parkinson’s disease (PD). However, little knowledge exists about the relation between modality effect and delayed recall impairment in PD. The primary goal of this study was to compare delayed free recall performance between three different memory modalities (verbal, visual, and cross visual-verbal) in a sample of non-demented patients with mild PD progression. The secondary goal was to explore the frequency of deficient performance on the basis of normative comparisons on each of the three delayed free-recall measures.

**Method**: A total of 71 non-demented patients with mild PD progression were recruited for the administration of the Montreal Cognitive Assessment (MoCA), the Rey Auditory Verbal Learning Test (RAVLT), the Rey Osterrieth Complex Figure Test (ROCFT), and the Greek Version of Face-Name Associative Memory Examination (GR-FNAME12).

**Results**: The percentages of deficient-performances for the three delayed free recall measures were 45.1% (32/71), 39.4% (28/71) and 31% (22/71) for the GR-FNAME12, ROFCT and RAVLT, respectively. The results indicated no significant difference between performances of the GR-FNAME12 and ROCFT, both of which were significantly lower than performance on the RAVLT.

**Conclusions**: In conclusion, delayed free recall appears to be more severely affected in the cross visual-verbal and visual memory modalities than in verbal-memory modalities in the early phase of PD progression.

## Introduction

Parkinson’s disease (PD) is a neurodegenerative disease manifested by dopaminergic loss in the substantia nigra and characterized by frontal-striatal dysfunction (Davie, 2008). Tremor, rigidity, bradykinesia, and postural deformities are the clinical motor symptoms of this disease (Jankovic, 2008). However, PD is increasingly being recognized as a complex and systematic disease that also causes various non-motor changes, of which cognitive impairments are among the most common (Galvin, 2006). Cognitive status is among the primary predictive factors of quality of life and autonomous daily functioning. Thus, achieving a better understanding of how different components of cognitive functioning are impacted by PD can help clinicians identify strategies for enhancing cognitive status among patients exhibiting symptoms of decline.

The core neurocognitive profile of PD is associated with deficits on speed processing, attention, executive function, and visuospatial abilities (Pagonabarraga and Kulisevsky, 2012). In the domain of memory, studies have found mixed results. On the one hand, some research studies propose that patients with PD perform worse in delayed free recall tasks and better in recognition tasks (Higginson et al., 2005). These findings have formed the basis of a hypothesis that PD memory dysfunction is due to a failure in retrieval mechanisms associated with fronto-striatal pathology, which appears to reflect an executive deficit with secondary negative effect to the primary memory system (Tröster and Fields, 1995). In this context, patients with PD maintain the ability to access stored memories (recognition), while exhibiting difficulty or failure to initiate, organize and maintain efficient retrieval strategies (free recall). On the other hand, there are studies supporting that memory dysfunctions observed in non-demented patients could be a result of a deficit in the learning of new information (Chiaravalloti et al., 2014) or recognition (Whittington et al., 2000) because of hippocampal dysfunction. The findings of these studies have questioned the accuracy of the retrieval deficit hypothesis, thereby leaving the relevant research field open.

Nevertheless, regardless of the debate about the source of memory impairment in PD, previous studies have found that patients with PD score lower than non-afflicted individuals across various memory modalities (Ivory et al., 1999; Singh and Behari, 2006; Kormas et al., 2019); however, it has not yet been well-documented whether there is a tendency for non-demented patients with mild PD progression to perform differently in specific memory modalities. The objective of the current study was to address these questions by comparing the delayed free recall performance between three different memory modalities, namely verbal, visual, and cross visual-verbal, in a sample of non-demented patients with mild PD progression. Furthermore, we computed the frequency of deficient performance based on the normative comparisons for each delayed free-recall measure.

## Methods and Measures

### Participants

Patients were recruited from the Neurological Clinic of Aeginition Hospital in the capital city of Greece, Athens. The inclusion criteria were: (a) diagnosis of idiopathic PD; (b) classification I and II on the Hoehn & Yahr scale (mild progression; Hoehn and Yahr, 1967); (c) a Montreal Cognitive Assessment (MoCA) score above 22; and (d) no psychiatric or other neurological history. The PD group comprised 71 (26 females) right-handed patients from the ages of 48–83 years (mean: 67.22, SD: 8.25) with 3–18 years of formal schooling (mean: 11.99, SD: 3.97). The mean score on MoCA was 24.82 (SD = 2.11). The ethics committee of Aeginition Hospital approved the study protocol that used the principles outlined in the Declaration of Helsinki. All participants were informed of the study’s purpose. They then signed a written informed consent form before they could participate in the study.

### Neuropsychological Measures

Each patient was individually evaluated during morning hours in the First Neurology Department of the National and Kapodistrian University of Athens at Aeginition Hospital. The evaluation of global cognitive status was conducted with the MoCA (Nasreddine et al., 2005) standardized in Greek (Konstantopoulos et al., 2016); delayed free recall was measured with the Rey Auditory Verbal Learning Test (RAVLT; Rey, 1964) standardized in Greek for the verbal memory domain (Messinis et al., 2016), with the Rey Osterrieth Complex Figure Test (ROCFT; Rey, 1941) standardized in Greek Test (Aretouli and Kosmidis, 2007) for the visual memory domain and with the Greek Version of Face–Name Associative Memory Examination (GR-FNAME12) for the cross visual-verbal memory domain (Kormas et al., 2018).

### Statistical Analysis

Statistical analysis of the resulting data was performed using SPSS23 (IBM Corp. Released 2015. IBM SPSS Statistics for Windows, Version 23.0. Armonk, NY, USA: IBM Corp.). Initially, each participant’s raw scores on the delayed recall measures were transformed into *z*-scores based on the age- and education-matched normative performance data of Greek general population. Thus, we used a standard score system, taking into account the effect of age and education. Next, a one-way analysis of variance (ANOVA) was used to compare the *z*-scores of the delayed free recall performances between the three different memory modalities. Finally, the frequency of deficient performance (*z*-score lower than −1.00) was estimated based on the normative comparisons.

## Results

**Figure 1:**
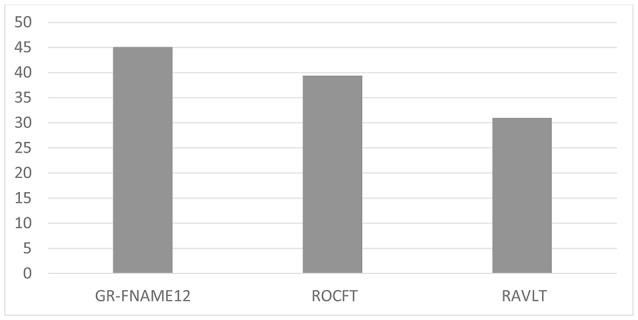
Percentage of deficient delayed free recall performance on three different memory modalities.

The results of the one-way ANOVA revealed significant differences in performance between the three memory modalities (*F*_(2,68)_ = 6.020, *p* < 0.05). The Bonferroni *post hoc* test demonstrated a significantly lower performance on the GR-FNAME12 (mean = −0.71, SD = 1.11) and ROCFT (mean = −0.27, SD = 1.28) compared with the RAVLT (mean = 0.04, SD = 1.48). In contrast, no significant difference was observed between performances of the GR-FNAME12 and ROCFT (Table 1). We found that the percentages of deficient-performance were respectively 45.1% (32/71), 39.4% (28/71), and 31% (22/71) for GR-FNAME12, ROFCT, and RAVLT (Figure 1).

**Table 1:** ANOVA comparisons of the *z*-scores of the delayed free recall performances on three different memory modalities.

				Bonferroni *Post hoc* comparisons		
Measures	*n*	Mean	SD	GR-FNAME12	ROCFT	RAVLT
GR-FNAME12	71	−0.71	1.11			
ROCFT	71	−0.27	1.28	0.131		
RAVLT	71	0.04	1.48	<0.05	<0.05	

## Discussion

In this study, we compared the *z*-scores of three distinct memory modalities measures in a sample of non-demented patients with mild PD progression. The main outcome of this research was that recall after delay among patients with PD is significantly lower for new material that is presented cross visually-verbally (face-name associations) and visually (complex figure) in comparison with material presented verbally (word list). We also found that a remarkable percentage of our study participants presented higher frequencies of deficient delayed free recall performance on the GR-FNAME12 and ROFCT than on the RAVLT.

This tendency of the modality effect could be analyzed in the view of the interaction between cognitive load of memory tasks and the executive deficits that characterize early-stage PD (Dirnberger and Jahanshahi, 2013). The GR-FNAME12 requires forming and recalling unfamiliar face-name associations, whereas the ROFCT demands recall of a complicated figure drawing. The meaninglessness of the names, the lack of semantic associations between faces and names and the abstract/nonsense nature of complex figure might negatively impact the retrieval mechanism by demanding more cognitive effort, greater attention resources, and the generation of more sophisticated internal strategies. In contrast, the RAVLT merely requires recalling a list of nouns. It seems that patients with early-stages PD can more efficiently retrieve words as supported by relatively intact semantic networks and the application of phonological or conceptual strategies.

The results of present study align with previous findings concerning visuospatial impairments in early-stage PD (Davidsdottir et al., 2005). Visuospatial deficits in PD are caused by a dysfunction in circuits between the basal ganglia, posterior parietal cortex and visual cortex (Middleton and Strick, 2000). These deficits include difficulties from basic to high-order cognitive visuospatial functions, which might negatively impact performance on various tasks that depend on visuospatial abilities (Cronin-Golomb and Amick, 2001). Both the GR-FNAME12 and ROFCT rely heavily on visuospatial processing compared with the RAVLT. In this context, PD patients retrieve new material in the visual modality with greater difficulty and effort.

The present study has some limitations. This was a cross-sectional research and the present data reflect task performances at a certain point in time. The patient selection criteria were based on the MoCA test for cognitive status and the Hoehn & Yahr scale for motor symptoms progression. Neuroimaging or biomarkers data for the patients of our sample were not available. Moreover, our study clinically classified the PD patients with regard to only the motor symptom progression based on the Hoehn & Yahr scale. Thus, the current experimental design was not able to investigate the effect on delayed recall performance of different symptoms of PD. Moreover, our study explored the modality effect only on retrieval mechanism but not for learning or recognition functions. Finally, our results represent the group of patients with mild PD progression and may not be generalized in patients with moderate or severe progression of symptoms.

In conclusion, the results of the present study suggest that non-demented patients with mild PD progression perform relatively poorly on the cross visual-verbal and visual modalities of delayed free-recall measures. Conversely, they retrieve new material from the verbal memory storage system more efficiently. These findings could be applied in rehabilitation interventions and treatment protocols for the development of compensation strategies or retraining tasks.

## Ethics Statement

The ethics committee of Aeginition Hospital approved the study protocol using the principles outlined in the Declaration of Helsinki. All participants were informed of the purpose of the study. They then signed a written informed consent form before they could participate in the study.

## Author Contributions

CK contributed to study concept and design, acquisition of data, analysis and interpretation of data and drafting of the manuscript. IZ, IE, EK and CP contributed to study concept and design and critical revision of the manuscript for important intellectual content.

## Conflict of Interest Statement

The authors declare that the research was conducted in the absence of any commercial or financial relationships that could be construed as a potential conflict of interest.
